# Transcriptomics-based liquid biopsy panel for early non-invasive identification of peritoneal recurrence and micrometastasis in locally advanced gastric cancer

**DOI:** 10.1186/s13046-024-03098-5

**Published:** 2024-06-28

**Authors:** Ping’an Ding, Haotian Wu, Jiaxiang Wu, Tongkun Li, Renjun Gu, Lilong Zhang, Peigang Yang, Honghai Guo, Yuan Tian, Jinchen He, Jiaxuan Yang, Ning Meng, Xiaolong Li, Lingjiao Meng, Qun Zhao

**Affiliations:** 1 The Third Department of Surgery, the Fourth Hospital of Hebei Medical University, Shijiazhuang, 050011 China; 2 Hebei Key Laboratory of Precision Diagnosis and Comprehensive Treatment of Gastric Cancer, Shijiazhuang, 050011 China; 3 Big Data Analysis and Mining Application for Precise Diagnosis and Treatment of Gastric Cancer Hebei Provincial Engineering Research Center, Shijiazhuang, 050011 China; 4 School of Chinese Medicine, Nanjing University of Chinese Medicine, Nanjing University of Chinese Medicine, Nanjing, Jiangsu, 210023 China; 5 Department of Gastroenterology and Hepatology, Jinling Hospital, Medical School of Nanjing University, Nanjing, Jiangsu 210002 China; 6 Department of General Surgery, Renmin Hospital of Wuhan University, Wuhan, Hubei 430065 China; 7 Department of General Surgery, Shijiazhuang People‘s Hospital , Shijiazhuang, Hebei 050050 China; 8 Department of General Surgery, Baoding Central Hospital, Baoding , Hebei, 071030 China; 9 Research Center and Tumor Research Institute of the Fourth Hospital of Hebei Medical University, Shijiazhuang, 050011 China

**Keywords:** Gastric cancer, Peritoneal recurrence, Micrometastasis, Liquid biopsy, Transcriptomics panel

## Abstract

**Background:**

This study aimed to develop a novel six-gene expression biomarker panel to enhance the early detection and risk stratification of peritoneal recurrence and micrometastasis in locally advanced gastric cancer (LAGC).

**Methods:**

We used genome-wide transcriptome profiling and rigorous bioinformatics to identify a six-gene expression biomarker panel. This panel was validated across multiple clinical cohorts using both tissue and liquid biopsy samples to predict peritoneal recurrence and micrometastasis in patients with LAGC.

**Results:**

Through genome-wide expression profiling, we identified six mRNAs and developed a risk prediction model using 196 samples from a surgical specimen training cohort. This model, incorporating a 6-mRNA panel with clinical features, demonstrated high predictive accuracy for peritoneal recurrence in gastric cancer patients, with an AUC of 0.966 (95% CI: 0.944–0.988). Transitioning from invasive surgical or endoscopic biopsy to noninvasive liquid biopsy, the model retained its predictive efficacy (AUC = 0.963; 95% CI: 0.926–1.000). Additionally, the 6-mRNA panel effectively differentiated patients with or without peritoneal metastasis in 95 peripheral blood specimens (AUC = 0.970; 95% CI: 0.936–1.000) and identified peritoneal micrometastases with a high efficiency (AUC = 0.941; 95% CI: 0.874–1.000).

**Conclusions:**

Our study provides a novel gene expression biomarker panel that significantly enhances early detection of peritoneal recurrence and micrometastasis in patients with LAGC. The RSA model's predictive capability offers a promising tool for tailored treatment strategies, underscoring the importance of integrating molecular biomarkers with clinical parameters in precision oncology.

**Supplementary Information:**

The online version contains supplementary material available at 10.1186/s13046-024-03098-5.

## Background

Gastric cancer is the second leading cause of cancer-related mortality globally [1, 2]. It predominantly manifests in advanced stages, leading to a substantial proportion of patients being initially diagnosed with metastatic disease or recurrence after adjuvant therapy [3–5]. Peritoneal dissemination is the primary mechanism of recurrence and distant metastasis in locally advanced gastric cancer (LAGC), ultimately leading to poor prognosis, with a median survival of less than 12 months [6, 7]. The ineffectiveness of conventional systemic chemotherapy and limited therapeutic alternatives is chiefly responsible for the unfavorable outcomes in patients with peritoneal metastases (PM) from gastric cancer [8, 9]. Additionally, the absence of robust diagnostic tools for the early detection of PM constitutes a significant barrier to enhancing patient outcomes. Currently, staging laparoscopy is increasingly utilized because of its superior diagnostic precision in identifying PM in gastric cancer patients compared to computed tomography (CT) or positron emission tomography CT (PET-CT) scans [10–13]. Despite its advantages, the broader adoption of staging laparoscopy as a standard practice is impeded by its invasive nature, necessity for general anesthesia, and comparatively higher costs [10]. While numerous retrospective studies have underscored the importance of staging laparoscopy for detecting PM, consensus on its routine application in patients with LAGC remains elusive [14–17].

An increasing number of studies have established a link between the presence of intraperitoneal cancer cells and an increased occurrence of peritoneal recurrence or metastasis in gastric cancer patients [18]. The 8th edition of the American Joint Committee on Cancer (AJCC)/ International Union Against Cancer Classification (UICC) adopts washing cytology to detect malignant cells, thereby facilitating more precise tumor staging [19]. Tumors that are cytology-positive, even in the absence of visible metastases (P0CY1), are classified as advanced cancers with distant metastasis [20, 21]. Early identification of intraperitoneal cancer cells is essential because it necessitates a distinct treatment approach that significantly enhances patient outcomes [22]. Preoperative detection of PM allows for the implementation of aggressive treatments such as neoadjuvant intraperitoneal and systemic chemotherapy (NIPS), hyperthermic intraperitoneal chemotherapy (HIPEC), and cytoreductive surgery (CRS) [23–27]. Although these treatments have not been standardized, emerging evidence underscores their potential to significantly improve the prognosis. This highlights the importance of detecting metastatic tumors at an early stage and the critical role of the early detection of peritoneal dissemination in improving treatment efficacy. Therefore, the development of accurate molecular biomarkers for the early detection of PM has the potential to significantly lower the morbidity and mortality associated with this condition through timely interventions.

Currently, no molecular biomarkers are clinically available for the detection of PM. While conventional tumor markers such as CA125, CA19-9, and CA72-4 are often upregulated in patients with LAGC and overexpression in the sera of patients with PM has been reported, the diagnostic accuracy of using these individual biomarkers or in combination remains insufficient to detect PM [28–30]. Additionally, obtaining cytological specimens through invasive abdominal lavage under general anesthesia is challenging because of the procedure's low sensitivity, interobserver variability, and limited ability to distinguish well-differentiated cancer cells from normal mesothelial cells [31–33]. The development of robust molecular biomarkers for identifying gastric cancer patients at high risk of peritoneal recurrence or metastasis could be clinically transformative. This advancement would enable timely intervention, potentially revolutionizing clinical practice.

Recent advances in next-generation sequencing have enabled multi-omics analyses to identify potential molecular biomarkers for PM in gastric cancer [34, 35]. However, previous studies have had limitations, including incomplete biomarker discovery and validation methods, small cohort sizes, and lack of independent validation cohorts, hindering clinical translation [36–41]. Here, we addressed these limitations by performing systematic genome-wide transcriptome profiling of gastric cancer tissue specimens, followed by rigorous bioinformatics analysis to identify gene markers predicting peritoneal recurrence post-surgery and adjuvant therapy. We subsequently developed a risk prediction model using these genetic markers that was successfully validated to predict peritoneal recurrence in a multicenter cohort of surgical, endoscopic, and blood specimens from patients with gastric cancer, transitioning to a non-invasive liquid biopsy approach. Importantly, given the association between peritoneal recurrence and metastases, we evaluated the diagnostic performance of our gene panel for detecting PM using multiple independent prospective cohorts of patients with initial metastases or P0CY1 tumors. Through this systematic and comprehensive biomarker discovery and multi-specimen validation approach, we identified a six-gene panel and risk stratification model to detect high peritoneal carcinomatosis risk, which, if identified early, could inform clinical decisions and improve patient outcomes.

## Methods and materials

### Biomarker discovery in genome-wide expression profiling datasets

**Fig. 1:**
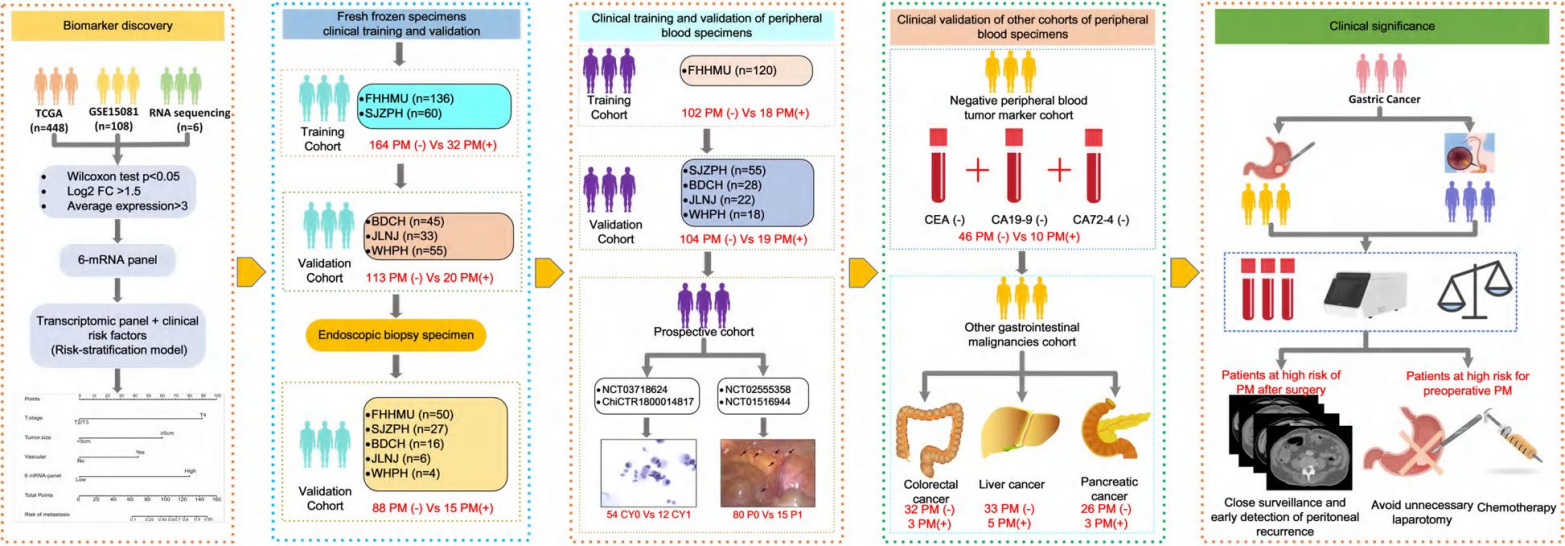
Flowchart of study design for discovery and validation of 6-mRNA markers in different LAGC patient populations

The biomarker discovery and validation processes used in this study are shown in Fig. 1. Initially, mRNA sequencing data from multiple sources, including the Gene Expression Omnibus (GEO) database [https://www.ncbi.nlm.nih.gov/geo/, GSE15081], the Cancer Genome Atlas (TCGA) database, and matched specimens with PM recurrence, were employed for biomarker identification..

We initially evaluated the expression of selected mRNAs using real-time quantitative polymerase chain reaction (RT-qPCR) in a pilot cohort of 29 matched GC and adjacent non-malignant (ANM) tissue samples. Additionally, we collected peripheral blood specimens from 22 patients diagnosed with gastric cancer and 22 healthy individuals who underwent physical examinations within the same timeframe to examine the expression of candidate mRNA in the bloodstream. The samples were acquired from the Fourth Hospital of Hebei Medical University (FHHMU) between January and March 2023. Supplementary Table 1 presents the findings from these cohorts, including the clinicopathological details.

### Clinical cohorts for biomarker validation

This study involved the collection of 329 fresh frozen specimens from patients with LAGC across two independent cohorts, as detailed in Supplementary Table 2, for the development and validation of biomarkers predictive of PM recurrence. Specimens were collected from August 2016 to March 2019, excluding patients who received neoadjuvant therapy or had residual stomach cancer following partial gastrectomy. The training cohort, comprising 196 patients, was assembled from the Fourth Hospital of Hebei Medical University (FHHMU) and Shijiazhuang People's Hospital (SJZPH), while the validation cohort of 133 patients was assembled from Baoding Central Hospital (BDCH), Nanjing University Jinling Hospital (JLNJ), and Wuhan University People's Hospital (WHPH).

Further analysis included 103 matched gastroscopic biopsy specimens from the LAGC patient cohort across five institutions to validate the biomarker shift from surgical to gastroscopic biopsy specimens. The clinical characteristics of the cohorts are presented in Supplementary Table 3.

Additionally, serum samples from patients with LAGC, including those with and without post-surgery PM recurrence, were retrospectively analyzed to identify tissue-based biomarkers for liquid biopsy assays. The training cohort comprised 120 patients with LAGC treated between February 2017 and December 2019 at FHHMU, and the validation cohort included serum samples from 123 patients with LAGC from January 2016 to December 2019 across the four other institutions. The same inclusion and exclusion criteria were applied to the fresh frozen specimen cohorts, with detailed clinicopathological data in Supplementary Table 4.

Additionally, an analysis was conducted to assess the effectiveness of the biomarker panel in detecting PM and micrometastases in patients with gastric cancer. Peripheral blood specimens from 66 patients with LAGC, including 12 with confirmed P0CY1 status by peritoneal cytology (FHHMU, registration: NCT03718624, ChiCTR1800014817), and 95 patients with LAGC, including 15 confirmed PM patients by laparoscopic exploration from two other prospective studies (FHHMU, registration: NCT02555358, NCT01516944), were examined to predict P0CY1 occurrence and PM onset, respectively. The clinical information of these patients is summarized in Supplementary Table 5.

Furthermore, the specificity of our mRNA panel as a biomarker for PM in LAGC was evaluated against other gastrointestinal malignancies, including colorectal, pancreatic ductal, and hepatocellular carcinomas, using RT-qPCR on serum samples collected between 2019 and 2021 from patients with FHHMU.

Patient follow-up for recurrence or disease progression was conducted via laboratory tests, endoscopy, and abdominopelvic CT, adhering to the gastric cancer treatment guidelines. Tissue specimens were immediately frozen in liquid nitrogen and stored at -80 °C. Surgical specimens were processed according to the Chinese Society of Clinical Oncology guidelines. Tumor and lymph node staging was performed according to the 8th AJCC edition. All procedures were performed in accordance with the Declaration of Helsinki, and written informed consent was obtained from all participants and approval was obtained from the institutional review boards of all involved institutions.

### RNA extraction and gene expression analysis

We isolated total RNA from fresh-frozen surgical tissues using TRIzol reagent (Invitrogen, Frederick, MA, USA) according to the manufacturer's protocol [42]. For serum samples, we used the PAXgene Blood RNA Kit (Qiagen, Hilden, Germany) Total RNA was extracted [43]. Next, total RNA was reverse transcribed into cDNA using the GoScript Reverse Transcription System Kit (Promega) according to the manufacturer's instructions. Finally, we performed qRT-PCR analysis on the samples [44]. Relative target gene abundance was determined using the 2^^−ΔΔCT^ method and normalized to the GAPDH internal control, where ΔCT is the difference between the target and GAPDH CT values. Specific PCR primers used are listed in Supplementary Table 6.

### PPI protein interaction network analysis, pathway analysis and chemotherapy drug sensitivity analysis

We used the STRING database (https://string-db.org) to construct the Protein–Protein Interaction (PPI) network for Homo sapiens. The six-gene candidate list was analyzed using Enrichr (https://maayanlab.cloud/Enrichr/) for gene set and pathway analyses [45]. OncoPredict, predicted drug responses and biomarker efficacy using cell-line screening data [46]. Using the Genomics of Drug Sensitivity in Cancer (GDSC) database (https://www.cancerrxgene.org/), we identified potential therapeutic drugs for each gene and used the Wilcoxon rank-sum test to assess drug sensitivity differences between groups.

### Statistical analysis

Statistical analyses were performed using IBM SPSS version 23, R version 3.6.3, and GraphPad Prism version 8.0. Univariate and multivariate logistic regression analyses identified significant clinicopathological variables and mRNA classifiers as covariates; variables significant in univariate analysis were subsequently included in the multivariate regression. During the discovery phase, differential gene expression between the PM recurrence and non-PM recurrence groups was examined using the Wilcoxon rank sum and Bonferroni tests. The clinical validation phase involved modeling gene-based risk scores via logistic regression employing backward elimination, with model performance evaluated using receiver operating characteristic (ROC) curves and area under the curve (AUC) values. AUCs were derived from the ROC curves using the pROC package in R, and ROC curve comparisons were performed using the DeLong test. Sensitivity, specificity, positive predictive value (PPV), negative predictive value (NPV), precision, and accuracy for the 6-mRNA biomarker sets across all cohorts were determined using the report ROC package displayed in a confusion matrix. The optimal cutoff for ROC curves was established using the Youden index in the pROC package. Recurrence prediction and metastasis detection were facilitated by categorizing individuals into high- or low-risk groups based on the Youden index and median risk score, respectively. Recurrence-free survival (RFS) was analyzed using the Kaplan–Meier method, defined as the interval from surgery to peritoneal recurrence confirmation or death from any cause, with a 5-year review for recurrence-free patients who were alive at this milestone. Patients who were lost to follow-up without evidence of recurrence before 5 years were assessed at their last visit. Statistical significance was set at a *P*-value of < 0.05.

## Results

### Genome-wide gene expression profiling identification of overexpressed candidate mRNA in patients with peritoneal recurrence of GC

**Fig. 2:**
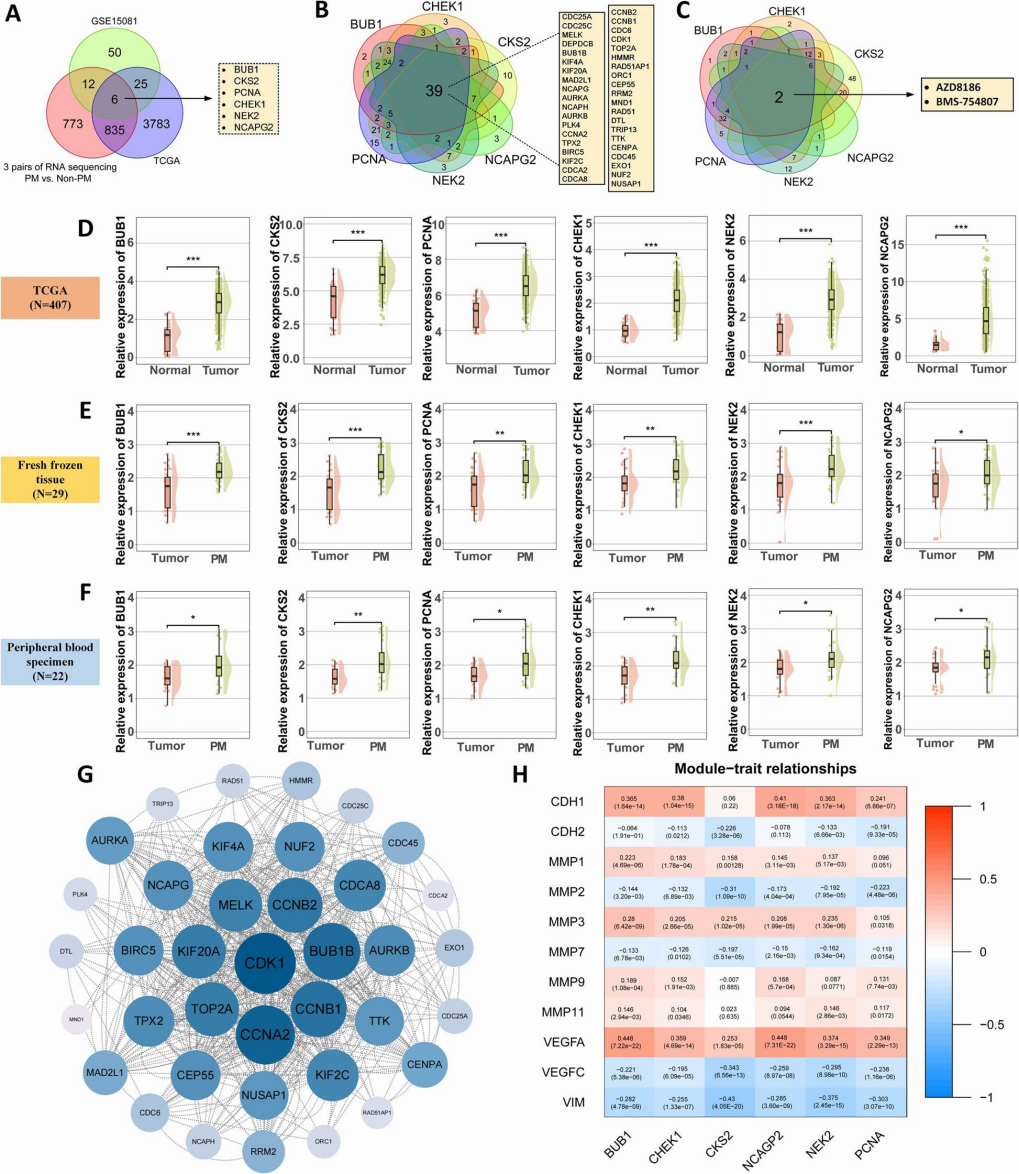
Transcriptomics-based discovery process and preliminary validation of candidate markers for peritoneal recurrence in LAGC patients. **A** Six candidate mRNAs were discovered using appropriate transcriptome data from the TCGA database, the GEO database, and paired mRNA sequencing Venn diagrams of 3 pairs with and 3 pairs without peritoneal recurrence. **B** The related genes of the six genes in the panel were intersected, and 39 common related genes were listed. **C** The 6 mRNA pair GDSC database contains drug sensitivity analysis Venn diagrams for 198 anticancer drugs. **D** Comparison of the expression of six mRNA (BUB1, CKS2, PCNA, CHEK1, NEK2, NCAPG2) in cancer lesions and normal tissues in the TCGA database. **E** Comparison of the expression of 6 mRNA in cancer lesions and normal tissues in 29 cases of fresh frozen tissues. **F** Comparison of 6 mRNA expressions in peripheral blood samples of 22 GC patients and healthy people. **G** Use the online STRING database (https://string-db.org) to construct the PPI network of these 6 mRNA. **H** Analysis of correlation heat map between 6 mRNA and common transferred genes based on TCGA database

In this study, we conducted an initial biomarker discovery by analyzing transcriptomic data from two publicly available gastric cancer datasets (TCGA and GSE15081), complemented by mRNA sequencing from three pairs of gastric cancer tissues with peritoneal metastasis recurrence post-radical surgery and three pairs without. The GSE15081 dataset comprised of 33 patients with peritoneal recurrence and 75 patients without recurrence. Through differential gene expression analysis (Wilcoxon rank sum test for GSE15081 and EdgeR for TCGA, both *P* < 0.05, with Bonferroni correction applied) and correlation analysis (*r* < 0.5), we identified six genes (BUB1, CKS2, PCNA, CHEK1, NEK2, and NCAPG2) that were differentially expressed between patients with and without peritoneal recurrence (Fig. 2A).

Further analysis of TCGA data revealed that these genes were significantly overexpressed in cancerous tissues compared to ANM (*P* < 0.05) (Fig. 2D), a finding consistent across pan-cancer analyses in TCGA database (Supplementary Fig. 1A-F). To validate these results, we established a pilot cohort at FHHMU, comprising gastric cancer and a health-screening population from January to December 2023. Analysis of 29 matched GC and PM tissue samples via RT-qPCR confirmed the higher expression levels of these mRNAs in PM tissues (*P* < 0.05) (Fig. 2E). Peripheral blood analysis of 22 gastric cancer patients and 22 PM patients from the same period also demonstrated elevated expression of the candidate genes in PM patients (Fig. 2F).

To investigate the potential association between gene expression in our panel and drug sensitivity, we employed the "oncoPredict" R package. This software analyzes expression matrices alongside drug response data from the Genomics of Drug Sensitivity in Cancer (GDSC) database, encompassing information on 198 anticancer drugs. Analysis of the GSE15081 dataset revealed that the groups with high expression of the six identified genes exhibited increased sensitivity to BMS-754807 and AZD8186 (Fig. 2C**, **Supplementary Fig. 2A-M).

Pathway analysis of these genes was conducted using Enrichr, focusing on KEGG and GO enrichment, and the results are presented in Supplementary Fig. 1G. Through KEGG enrichment analysis, we found that the related genes of the six genes are involved in regulating signaling pathways such as "cell cycle", "p53 signaling pathway", and "cell senescence". In addition, the results show that CKS2, PCNA, CHEK1, and NCAPG2 are also involved in the regulation of tumor DNA replication and mismatch repair.

The results of GO enrichment analysis showed that BUB1, PCNA, CHEK1, NEK2, and NCAPG2 are all related to single-stranded DNA binding. BUB1, CHEK1, NEK2, and NCAPG2 are also involved in the biological process of DNA replication origin binding. In addition, we found that BUB1, CKS2, CHEK1, NEK2, and NCAPG2 are involved in regulating cell mitosis processes such as microtubule binding and tubulin binding. At the same time, BUB1, CKS2, PCNA, CHEK1, NEK2, and NCAPG2 all regulate the biological processes of serine/threonine/tyrosine kinase activity. In terms of cellular components and molecular functions, the results show that the six gene-related genes are located in various cell division-related cellular components such as the spindle, microtubule cytoskeleton, and mitotic spindle. They also regulate mitotic sister chromatid separation, mitotic spindle Cell division related molecular functions such as organization and organization of microtubule cytoskeleton involved in mitosis.

Through the results of KEGG and GO enrichment analysis, it was found that the six genes are involved in regulating the cell cycle, especially related to cell mitosis. The results show that the six genes are related to the division of cancer cells in gastric cancer and may affect the occurrence and development of gastric cancer by regulating the cell cycle and aging of cancer cells, thus affecting the peritoneal metastasis of gastric cancer.

Protein interaction networks were mapped using the STRING database and visually analyzed with Cytoscape 3.9.1 (Fig. 2B, G), elucidating their potential roles in gastric cancer. Moreover, correlation analyses with metastasis-related genes (MMP1, MMP3, and VEGFA) via Timer 2.0 (http://timer.cistrome.org/) showed positive associations, with additional correlations detailed in Fig. 2H.

### Validation of the 6-mRNA panel of surgical resection specimens to predict postoperative peritoneal recurrence in patients with LAGC

**Fig. 3:**
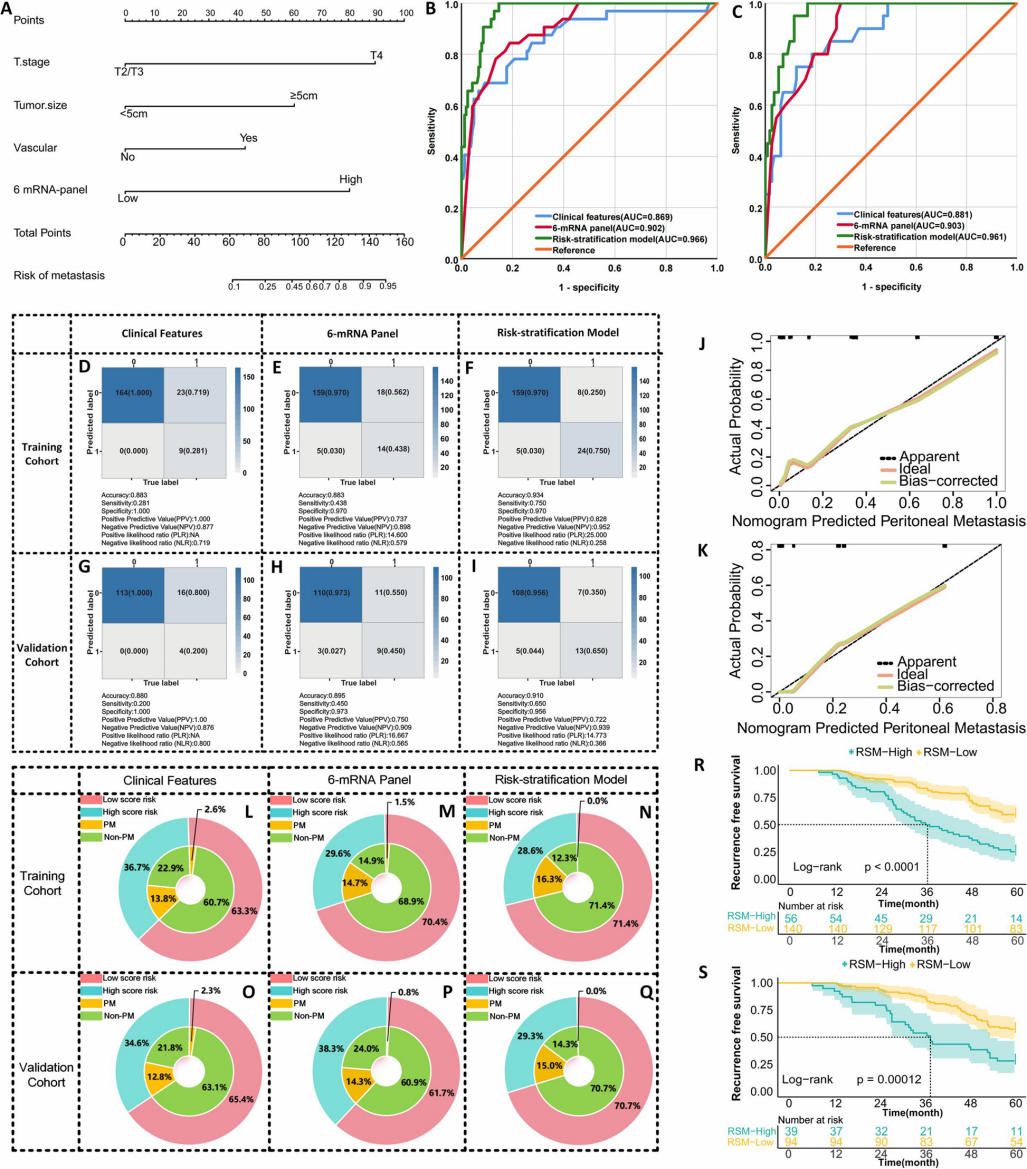
Transcriptome training and validation phase for identifying peritoneal recurrence in surgical specimens from LAGC patients. **A** Nomogram of peritoneal recurrence in LAGC patients constructed based on 6-mRNA combined with clinical characteristics; (**B**) ROC curve of different predictor variables in the training set. **C** ROC curve for different predictors in the validation set. **D**-**I** Prediction confusion matrix plots for RSA models built using clinical features, 6-mRNA, and both in the training and validation sets. **J** Calibration curve of the RSA model in the training set. **K** Calibration curve of the RSA model in the validation set. **L**-**Q** Clinical benefit plots in the training set and validation set of the RSA model constructed using clinical features, 6-mRNA and their combination. **R** Log-rank test survival curve plot of the training set patients divided into low-risk and high-risk groups based on the critical value derived from the Youden index of the nomogram. **S** Log-rank test survival curve plot that divides patients in the validation set into low-risk and high-risk groups based on the critical value derived from the Youden index of the nomogram

We evaluated the efficacy of the 6-mRNA panel by RT-qPCR in a training cohort of 164 patients with LAGC without and 32 with peritoneal recurrence post radical resection. Binary logistic regression was used to assess the predictive ability of mRNA levels for peritoneal recurrence. Multivariate analysis showed that each gene independently affected peritoneal recurrence risk (all *P* < 0.05, Supplementary Table 7). ROC curve analysis was used to determine individual and combined biomarker accuracy in distinguishing peritoneal recurrence cases. Although the individual mRNA markers were effective, the 6-mRNA panel showed superior diagnostic performance (AUC = 0.902, 95% CI: 0.851–0.953, *P* < 0.001; Fig. 3B). Further clinical evaluation of the 6-mRNA classifier included analysis of its ability to detect peritoneal recurrence alongside clinical variables. Multivariate analysis showed that the 6-mRNA classifier (OR = 6.634, 95% CI: 1.325–28.943, *P* = 0.015), tumor size (OR = 5.780, 95% CI: 1.183–30.873, *P* = 0.021), invasion depth (OR = 7.453, 95% CI: 1.569–35.401, *P* = 0.012), and vascular tumor thrombus invasion (OR = 4.124, 95% CI: 1.078–15.773, *P* = 0.038) were significant independent peritoneal recurrence indicators for LAGC postoperative complications (Supplementary Table 8).

We estimated the probability of peritoneal recurrence by applying a formula based on logistic regression coefficients and constants: [ (3.665 × 6-mRNA panel) + (5.009 × depth of invasion) + (2.009 × tumor size) + (1.417 × vascular tumor thrombus invasion) + (-12.340)], which is depicted as a nomogram for visualizing peritoneal recurrence predictions (Fig. 3A). Following calibration of this model with data from the training cohort, identical statistical parameters were applied to the validation cohort. This process enabled the stratification of patients into low- and high-risk categories based on cutoff values determined by the Youden index. Integrating the 6-mRNA panel with clinical variables, we developed a risk stratification assessment (RSA) model, which demonstrated superior predictive capability for peritoneal recurrence, as evidenced by an AUC of 0.966 (95% CI: 0.944–0.988, *P* < 0.001, Figs. 3D-F). Notably, according to the DeLong test, the RSA model had higher AUC values in the training set than the clinical model (0.966 vs. 0.869; *P* = 0.001) and 6-mRNA panel model (0.966 vs. 0.902; *P* = 0.038). The calibration curve of the model further underscored its exceptional predictive accuracy (Fig. 3J).

The model, retaining consistent statistical parameters, was subsequently applied to an independent external validation cohort of 133 patients with LAGC, comprising 20 patients with peritoneal recurrence and 113 patients without recurrence. This application underscored the robust predictive capacity of the RSA model, as evidenced by an AUC of 0.961 (95% CI: 0.931–0.991, *P* < 0.001) (Fig. 3C). Within this cohort, the RSA model demonstrated unparalleled sensitivity (65.0%) and specificity (95.6%), surpassing both the clinical model (sensitivity 20.0%, specificity 100.0%) and the 6-mRNA panel model (sensitivity 45.0%, specificity 97.3%) in terms of predictive performance (F igs. 3G-I). Calibration curve analysis further corroborated the enhanced predictive accuracy **(**Fig. 3K**)**.

We further assessed the potential of the RSA model to enhance the cost-effectiveness of clinical decision-making. In our training cohort, using existing clinical parameters, 36.7% of patients were deemed to be at high risk for peritoneal recurrence, with the remaining 63.3% classified as low-risk. Subsequent follow-up indicated that only 13.8% (27 out of 196 cases) of the high-risk group and 2.6% (5 out of 196 cases) of the low-risk group experienced peritoneal recurrence. This outcome suggests that the initial risk classification based on clinical characteristics led to 22.9% of the patients receiving unnecessary intensive follow-up, whereas 2.6% were not closely monitored as needed (Fig. 3L). Conversely, when applying the 6-mRNA classifier to the same cohort, more accurate risk stratification emerged, with 29.6% classified as high-risk and 70.4% as low-risk. Peritoneal recurrence occurred in 14.7% (29 patients) of the high-risk group and 1.5% (3 patients) of the low-risk group **(**Fig. 3M**)**. The implementation of the RSA model significantly lowered the rate of excessive follow-up in the high-risk category to 12.3% (24 patients) **(**Fig. 3N**)**. This trend was consistent in the external validation cohort, where the RSA model markedly decreased unnecessary follow-up for high-risk patients and minimized missed diagnoses in the low-risk group compared to the other models **(**Fig. 3O-Q**)**. A combined analysis of both cohorts revealed that the RSA model improved the detection of peritoneal recurrence, increasing the rate from 37.3% to 54.7% (Supplementary Fig. 2N). This indicates a substantial improvement in clinical decision-making, reducing the likelihood of unnecessary interventions in high-risk patients and increasing detection in low-risk patients.

Furthermore, we conducted survival follow-up of the enrolled patients based on the high- and low-risk groups of the nomogram and found that among the patients in the training group, the 5-year RFS of the high-risk group was significantly worse than that of the low-risk group (25.0% vs. 59.3%, *P* < 0.0001). A similar difference also existed in the validation set patients (28.2% vs. 57.4%, *P* = 0.00012) (Fig. 3 R-S).

### Validation of 6-mRNA panel for prediction of peritoneal recurrence in patients with LAGC using gastroscopy biopsy specimens

**Fig. 4:**
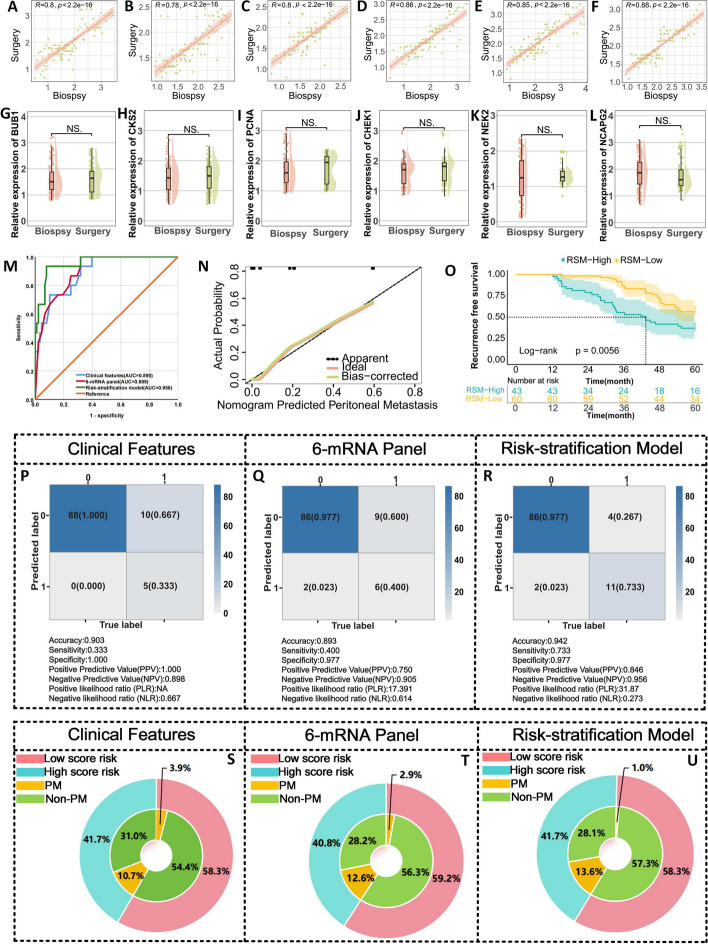
Transcriptome validation phase for identifying peritoneal recurrence in gastroscopic biopsy specimens from patients with LAGC. **A**-**F** Correlation analysis of six mRNAs in gastroscopic biopsy specimens and paired surgical resection specimens; (**G**-**L**) Comparison of the expression of six mRNAs in gastroscopic biopsy specimens and paired surgical resection specimens. **M** ROC curve for different predictor variables in gastroscopy biopsy specimens. **N** Calibration curve of the RSA model in gastroscopic biopsy specimens. **O** Log-rank test survival curve plot dividing these patients into low-risk and high-risk groups based on cutoff values derived from the Youden index of the nomogram. **P**-**R** Predictive confusion matrix plot jointly constructed by clinical features, 6-mRNA and RSA models. **S**-**U** Clinical benefit plot of the RSA model constructed using clinical features, 6-mRNA and their combination in the gastroscopy biopsy specimen validation set

In our study, beyond the surgically resected specimens from the training and validation cohorts, we acquired 103 matched endoscopic biopsy specimens, including 15 cases of peritoneal recurrence and 88 cases without recurrence. A notably high correlation was found in the expression profiles of six genes between biopsy and surgical specimens (Fig. 4A-F). A comparison of gene expression across these matched samples revealed no significant differences in the expression of any of the genes (Fig. 4G-L). The AUC value and calibration curve confirmed the validity and accuracy of the RSA model (Fig. 4M-N). Furthermore, within the biopsy cohort, the RSA model exhibited the highest sensitivity (73.3%) and specificity (97.7%) **(**Fig. 4P-R). Meanwhile, the RSA model enhanced the diagnostic rate of peritoneal recurrence in the high-risk group and reduced the diagnostic rate in the low-risk group (Fig. 4S-U). These findings underscore the capacity of the RSA model to refine clinical decision making and minimize unnecessary interventions.

As in the previous cohort follow-up, based on the high- and low-risk groups of the nomogram, we performed a log-rank test on patients with endoscopic biopsy specimens and found that the 5-year RFS of the high-risk group was significantly worse than that of the low-risk group (37.2% vs. 56.7%, *P* = 0.0056) (Fig. 4O).

### Validation of 6-mRNA panels in peripheral blood specimens to predict peritoneal recurrence in LAGC patients

**Fig. 5:**
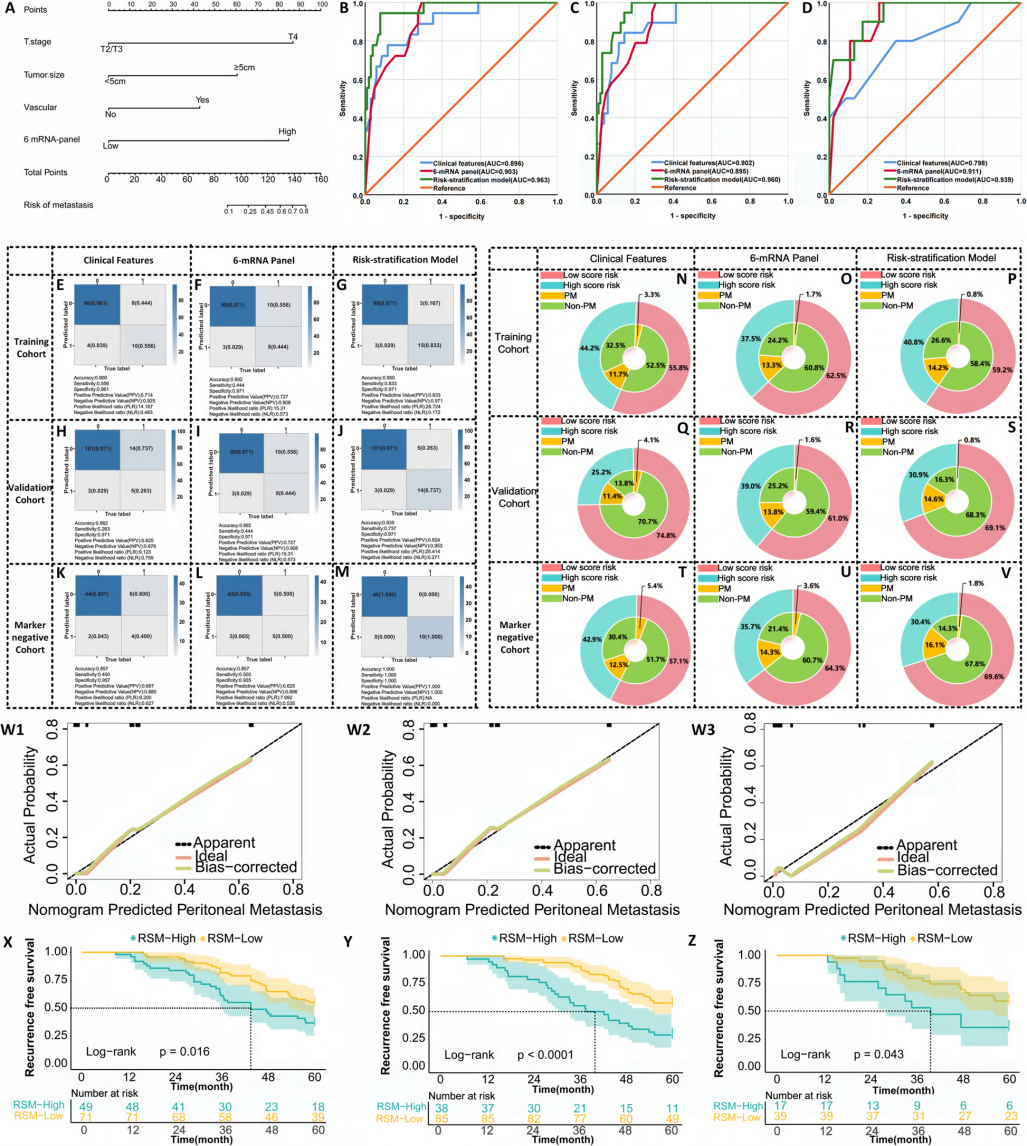
Transcriptome validation phase for identification of peritoneal recurrence in peripheral blood samples from LAGC patients. **A** Peritoneal recurrence nomogram of LAGC patients constructed based on 6-mRNA combined with clinical characteristics; (**B**) ROC curve of different predictor variables in the training set. **C** ROC curve for different predictors in the validation set. **D** ROC curve for different predictor variables in the tumor marker negative set. **E**-**M** Predictive confusion matrix plots of the RSA model built using clinical features, 6-mRNA, and both in the training, validation, and tumor marker negative sets. **N**-**V** Clinical benefit plots of the RSA model constructed using clinical features, 6-mRNA and their combination in the training set, validation set and tumor marker negative set. (W1) Calibration curve of the RSA model in the training set. (W2) Calibration curve of the RSA model in the validation set. (W3) Calibration curve of tumor marker negative concentrated RSA model. (X) Log-rank test survival curve plot of the training set patients divided into low-risk and high-risk groups based on the critical value derived from the Youden index of the nomogram. **Y** Log-rank test survival curve plot that divides patients in the validation set into low-risk and high-risk groups based on the critical value derived from the Youden index of the nomogram. **Z** Log-rank test survival curve plot that divides patients in the tumor marker-negative set into low-risk groups and high-risk groups based on the critical value derived from the Youden index of the nomogram

The primary objective of our study was to develop a liquid biopsy-based method to predict peritoneal recurrence in patients with LAGC. We measured mRNA expression levels in serum samples from 120 LAGC patients using a panel of 6 mRNAs. Logistic regression showed that each mRNA significantly affected the risk of peritoneal recurrence in LAGC patients (all *P* < 0.05, Supplementary Table 7). The prediction model built based on multivariable logistic regression had an AUC of 0.903 for the 6-mRNA panel (95% CI: 0.843–0.963, *P* < 0.001) (Fig. 5B), showing high prediction accuracy. Notably, the liquid biopsy RSA model outperformed the clinical and 6-mRNA models in predicting peritoneal recurrence (Fig. 5B). Risk stratification and calibration curve analysis confirmed its excellent performance (Fig. 5E-G, 5W1). Clinical benefit analysis showed that the RSA model improved the recurrence detection rate in high-risk patients and reduced the recurrence rate in low-risk patients (Fig. 5N-P). Follow-up results showed that the 5-year recurrence-free survival rate of the high-risk group was significantly lower than that of the low-risk group (Fig. 5X), emphasizing the potential of the RSA model in optimizing clinical decision-making and patient management.

An independent validation cohort of 104 non-relapsed and 19 relapsed LAGC patients was evaluated applying the same statistical model and coefficients originally used in the training cohort. The predictive ability of the RSA model was confirmed, with an AUC value of 0.960 (95% CI: 0.928–0.993, *P* < 0.001) (Fig. 5C). Confusion matrix and calibration curve analysis further confirmed the advantages of the RSA model compared with the 6-mRNA panel and clinical feature models (Fig. 5H-J, 5W2). These results demonstrate the effectiveness of tissue-based 6-mRNA panels successfully translated into liquid biopsy analysis. The RSA model improved the recurrence detection rate in the high-risk group and reduced the recurrence rate in the low-risk group (Fig. 5Q-S). After integrating the data from both the training and validation cohorts, the RSA model showed higher accuracy in predicting peritoneal recurrence (Supplementary Fig. 2O). The log-rank test showed that the 5-year recurrence-free survival rate in the high-risk group was significantly lower than that in the low-risk group (Fig. 5Y), underscoring the potential of the RSA model to improve clinical outcomes.

In current clinical practice, CA19-9, CA72-4, and CEA are used to monitor peritoneal recurrence in LAGC patients. We analyzed a cohort of 56 tumor marker-negative patients to evaluate the predictive ability of the RSA model. The RSA model was significantly better than the clinical characteristics and 6-mRNA models (Fig. 5D). Confusion matrix and calibration curve analysis support the high prediction accuracy of the RSA model (Figs. 5K-M, 5W3). Clinical benefit analysis showed that the RSA model was better at detecting peritoneal recurrence (Figs. 5K-M, 5W3). Moreover, survival analysis based on the nomogram's high- and low-risk categories revealed that, among patients negative for tumor markers, the 5-year RFS was significantly lower in the high-risk group than in the low-risk group (Fig. 5Z), emphasizing its potential in improving clinical decision-making and prognosis.

### 6-mRNA panel identifies the presence of peritoneal metastases and micrometastases in disease diagnosis of LAGC patients

**Fig. 6:**
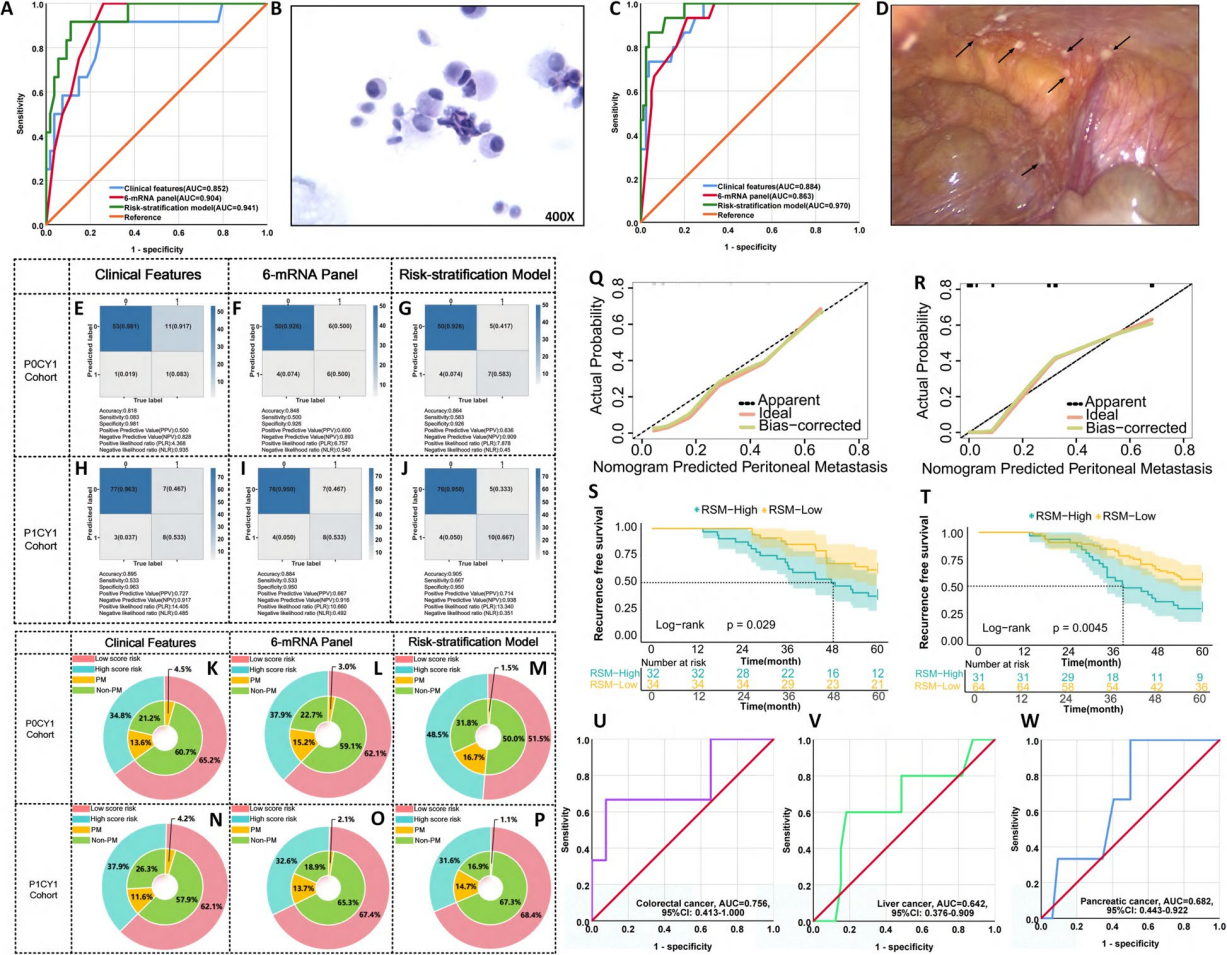
Transcriptome validation phase for identifying peritoneal metastases and micrometastases in peripheral blood samples from patients with LAGC. **A** ROC curve of different predictor variables predicting the occurrence of peritoneal micrometastases (P0CY1). **B** HE staining of free cancer cells shed from the peritoneal cavity. **C** ROC curve of different predictor variables predicting the occurrence of PM. **D** Diagnostic laparoscopy reveals peritoneal metastatic nodules. **E**-**G** Confusion matrix diagram for predicting the occurrence of peritoneal micrometastases (P0CY1) using clinical features, 6-mRNA and the RSA model constructed by the two. **H**-**J** Confusion matrix diagram for predicting the occurrence of peritoneal metastasis using an RSA model constructed using clinical features, 6-mRNA and their combination. **K**-**M** Clinical benefit plot for predicting the occurrence of peritoneal micrometastases (P0CY1) using clinical features, 6-mRNA, and a combined RSA model. **N**-**P** Clinical benefit plot for predicting the occurrence of PM using clinical features, 6-mRNA, and a combined RSA model. **Q** Calibration curve of the RSA model predicting peritoneal micrometastases in the (P0CY1) cohort. **R** Calibration curve of the RSA model predicting PM in the cohort. **S** Log-rank test survival curve plot for dividing peritoneal micrometastasis set (P0CY1) patients into low-risk and high-risk groups based on the critical value derived from the Youden index of the nomogram. **T** Log-rank test survival curve plot that divides patients in the PM set into low-risk and high-risk groups based on the critical value derived from the Youden index of the nomogram. **U** ROC curve of 6-mRNA predicting peritoneal metastasis of colorectal cancer. **V** ROC curve of 6-mRNA predicting peritoneal metastasis of hepatocellular carcinoma. **W** ROC curve of 6-mRNA predicting peritoneal metastasis of pancreatic ductal carcinoma

To evaluate the 6-mRNA panel for early detection of peritoneal metastases and micrometastases (P0CY1) in LAGC patients, we used a nomogram based on peripheral blood data. Despite current diagnostics often missing P0CY1 tumors, our RSA model, incorporating 6-mRNA and clinical features, confirmed its predictive power through studies (NCT03718624 and ChiCTR1800014817). Diagnostic laparoscopy identified P0CY1 in 12 of 66 patients, underscoring the need for novel therapies in this subgroup. Pre-surgical peripheral blood serum levels of the six mRNA analyzed via RT-qPCR revealed that the RSA model (AUC = 0.941) significantly surpassed both clinical features (AUC = 0.852) and the 6-mRNA panel (AUC = 0.904) in predicting P0CY1 (Fig. 6A, B). Furthermore, the confusion matrix and calibration curve analyses validated the superior prediction accuracy (Figs. 6E-G, 6Q). The RSA model increased detection of intraperitoneal micrometastases in high-risk patients from 13.6% to 16.7% and decreased misdiagnosis of peritoneal recurrence in low-risk patients from 4.5% to 1.5% (Figs. 6K-M). Stratifying patients by Youden index, follow-up revealed significantly lower 5-year RFS in the high-risk group versus the low-risk group (37.5% vs. 61.8%, *P* = 0.029) (Fig. 6S), highlighting the RSA model's potential to refine prognosis and guide decisions in LAGC.

Using serum from 95 LAGC patients in studies (NCT02555358 and NCT01516944), 15.8% developed PM post-laparoscopy. The RSA model (AUC = 0.970) outperformed clinical features (AUC = 0.884) and the 6-mRNA panel (AUC = 0.863) in predicting PM (Figs. 6C, D). Further analyses using confusion matrix plots and calibration curves confirmed the exceptional predictive performance (Figs. 6H-J, 6R). The RSA model improved PM detection in high-risk patients from 11.6% to 14.7% and reduced peritoneal recurrence misidentification from 4.2% to 1.1% (Figs. 6N-P). Follow-up showed a significant difference in 5-year RFS between high-risk (29.0%) and low-risk (58.3%) groups (*P* = 0.0045) (Fig. 6T). Our comprehensive biomarker research has yielded a novel gene prediction panel that improves gastric cancer management by enhancing peritoneal metastases detection, potentially boosting survival rates.

We assessed the 6-mRNA panel's diagnostic performance in LAGC serum samples and its efficacy in other gastrointestinal cancers: colorectal (*n* = 35), hepatocellular carcinoma (*n* = 38), and pancreatic adenocarcinoma (*n* = 29). The panel showed higher diagnostic accuracy for PM in LAGC (AUC = 0.879) compared to colorectal (AUC = 0.756), hepatocellular (AUC = 0.642), and pancreatic adenocarcinoma (AUC = 0.682) (Figs. 6U-W). DeLong's test confirmed its high specificity for LAGC over other gastrointestinal cancers (*P* < 0.001).

## Discussion

Recent studies have improved our understanding of the molecular heterogeneity of gastric cancer cells, but these insights have limited impact on clinical management [47–49]. Computed tomography, although effective in detecting larger metastatic lesions, fails to identify smaller lesions [50]. Although laparoscopy has high diagnostic accuracy, its invasiveness and cost limit its use, and it is mainly suitable for high-risk late-stage patients. The lack of precise molecular biomarkers to detect peritoneal recurrence and metastasis is a critical issue. This study identified a six-gene biomarker panel to predict peritoneal recurrence in gastric cancer patients and developed a risk stratification model (RSA model) to accurately distinguish high-risk patients, facilitate timely clinical intervention, and improve treatment outcomes. The results showed the effectiveness of the 6-mRNA biomarker panel in different samples, demonstrating its potential in clinical applications. The RSA model combining 6-mRNA panel with clinical parameters significantly helps identify gastric cancer patients with peritoneal metastasis.

In this study, we identified biomarkers associated with peritoneal recurrence by integrating mRNA sequencing data from two public databases and three paired samples. We identified six mRNAs that were significantly upregulated in peritoneal recurrence of LAGC and used the expression data and clinical characteristics of these mRNAs to construct an RSA model to predict peritoneal recurrence. This model showed high diagnostic accuracy (AUC = 0.966) in multicenter validation and was more effective than models based solely on clinical features. The high diagnostic accuracy of the 6-mRNA panel (AUC = 0.956) was further verified using paired endoscopic biopsy specimens, consistent with previous findings by Lee et al. [51]. Based on non-invasive liquid biopsy of peripheral blood, the RSA model maintained strong predictive power in training cohorts and multi-center validation, providing a valuable prognostic tool for LAGC patients with clinically negative tumor markers. Risk stratification according to the Youden index improves the predictive power of 5-year recurrence-free survival.

Additionally, we validated the model in other gastrointestinal cancers. Findings indicate that our 6-mRNA biomarker panel is highly specific for differentiating patients with LAGC from those with other gastrointestinal malignancies. However, except for gastric cancer, the AUC of other gastrointestinal cancers is not that low. Considering the functions of these 6 genes in cancer [52–57], this result indicates that these six genes may also play an important role in other gastrointestinal cancers and are general factors that enhance tumor invasion and metastasis. However, due to the small sample size, the results may also be statistically biased. We will further expand the sample size in future studies and conduct a prospective cohort study to analyze the role of these six genes in pan-cancer.

Previous studies have shown that liquid biopsy can increase the sensitivity of tissue biopsy. However, for cervical cancer, since most of the mRNA may exist in tissue, the detection performance of tissue may be more accurate than that of blood [58]. In the clinical cohort validation of this study, we found that the diagnostic performance of 6 mRNAs had high predictive value in both tissue and blood samples, but there was no significant difference. This may be due to the statistical bias produced by the small number of samples. On the other hand, it may be that the RSA model itself has high predictive performance. In addition, over the past decade, invasive technologies for diagnosing and monitoring cancer have been slowly being replaced by non-invasive technologies such as liquid biopsy due to their non-invasive nature, simplicity of operation, and ease of repeated sampling throughout the treatment process [59]. Therefore, peripheral blood-based biomarkers may be a preferable choice for accurately identifying the peritoneal recurrence risk or preoperative peritoneal metastasis, facilitating safer and more accessible sample collection for analysis without increasing the risk of invasive procedures.

The most effective treatment strategy for gastric peritoneal carcinomatosis remains elusive. Although systemic or intraperitoneal chemotherapy has been shown to enhance overall survival compared to symptomatic treatment alone, the prognosis for this condition remains poor [60]. Evidence from prior research suggests that for patients with limited metastases, multimodal therapy can offer superior long-term survival benefits and a more favorable prognosis than chemotherapy alone [61]. Our 6-mRNA panel has proven to be an effective tool for distinguishing between patients with P0CY1 tumors and those with visible carcinomatosis, thereby playing a crucial role in identifying candidates for conversion therapy.

### Limitations

Our study is subject to potential limitations stemming from its retrospective design, which might have introduced selection bias. Notably, the modest sample size, particularly the small number of patients with peritoneal recurrence, could undermine the robustness of the outcomes of our model. Consequently, this underscores the necessity for future prospective clinical trials involving a broader cohort to substantiate the diagnostic precision of our developed RSA model. Furthermore, although our investigation encompasses clinical cohorts from various institutions, the exclusive focus on Chinese patients with specific clinicopathological features limits the extrapolation of our findings to diverse populations. This limitation highlights the need for international multicenter studies with large sample sizes to comprehensively assess biomarker efficacy, thereby facilitating their integration into standard clinical practice and broadening the applicability of our conclusions. Additionally, our risk stratification model, which amalgamates mRNA and clinical parameters, must be evaluated in light of evidence linking elevated expression of common clinical markers (e.g., HER2, PDL1, Claudin18.2) and DNA mutations with increased peritoneal recurrence and micrometastasis risks. Given the preference for simpler clinical applications, future research should investigate these markers or DNA mutation status to enhance diagnostic accuracy for peritoneal recurrence. Despite these constraints, our research offers pivotal insights into peritoneal recurrence detection in patients with LA GC, potentially advancing the development of effective molecular biomarkers for the risk assessment and management of these lethal malignancies.

## Conclusions

In summary, through a rigorous discovery and validation process, we developed and validated a novel gene expression biomarker panel for the detection of peritoneal recurrence in patients with LAGC and demonstrated its efficacy across several independent clinical cohorts. Additionally, our research showed that the RSA model, integrating a 6-mRNA panel with clinical parameters, effectively predicts peritoneal recurrence in populations traditionally negative for biomarkers. Most crucially, our risk stratification model is capable of preemptively identifying both PM and micrometastases (P0CY1) in gastric cancer patients. This advancement holds significant potential for enhancing clinical staging accuracy and informing tailored treatment strategies, ultimately improving outcomes in patients with gastric cancer.

### Supplementary Information

Supplementary Material 1.

Supplementary Material 2.

Supplementary Material 3.

Supplementary Material 4.

Supplementary Material 5.

Supplementary Material 6.

Supplementary Material 7.

Supplementary Material 8.

Supplementary Material 9.

## Data Availability

The processed data in this study have been deposited in GEO under accession number GSE248612. The participant data with identifiers used to support the findings of this study were supplied by Qun Zhao under license and thus cannot be made freely available. The requests for access to these data should be made to Qun Zhao, zhaoqun@hebmu.edu.cn.
