# Supplementary material for: Transcriptomics-based liquid biopsy panel for early non-invasive identification of peritoneal recurrence and micrometastasis in locally advanced gastric cancer
Source: J Exp Clin Cancer Res. 2024 Jun 28;43:181. doi: 10.1186/s13046-024-03098-5 (PMC11212226; doi:10.1186/s13046-024-03098-5)
Supplement: Supplementary file 1 — Supplementary Material 1. [file 13046_2024_3098_MOESM1_ESM.docx]

**Supplementary Table 1 Clinical characteristics of cohorts examining candidate mRNA expression in surgical resection specimens and peripheral blood specimens**

| **Clinical characteristic** | **Surgical specimen cohort (N=29)** | **Peripheral blood specimen cohort (N=22)** |
| --- | --- | --- |
| **Gender** |  |  |
| Male | 16 (55.17) | 12 (54.55) |
| Female | 13 (44.83) | 10 (45.45) |
| **Age(years)** |  |  |
| ≤65 | 18 (62.07) | 12 (54.55) |
| ＞65 | 11 (37.93) | 10 (45.45) |
| **T stage** |  |  |
| T2/T3 | 20 (68.97) | 15 (68.18) |
| T4 | 9 (31.03) | 7 (31.82) |
| **N stage** |  |  |
| N0 | 7 (24.14) | 4 (18.18) |
| N+ | 22 (75.86) | 18 (81.82) |
| **Primary site** |  |  |
| Up 1/3 | 9 (31.03) | 7 (31.82) |
| Middle 1/3 | 7 (24.14) | 5 (22.73) |
| Lower 1/3 | 13 (44.83) | 10 (45.45) |
| **Tumor size(cm)** |  |  |
| ≤5 | 12 (41.38) | 10 (45.45) |
| ＞5 | 17 (58.62) | 12 (54.55) |
| **Histology** |  |  |
| None/Low | 20 (68.97) | 15 (68.18) |
| High/Median | 9 (31.03) | 7 (31.82) |
| **Lauren** |  |  |
| Diffuse/Mix type | 20 (68.97) | 17 (77.27) |
| Intestinal type | 9 (31.03) | 5 (22.73) |
| **Vascular invasion** |  |  |
| Yes | 19 (65.52) | 6 (27.27) |
| No | 10 (34.48) | 16 (72.73) |
| **Nerve invasion** |  |  |
| Yes | 15 (51.72) | 12 (54.55) |
| No | 14 (48.28) | 10 (45.45) |
| **BUB1** |  |  |
| Low | 11 (37.93) | 8 (36.36) |
| High | 18 (62.07) | 14 (63.64) |
| **CKS2** |  |  |
| Low | 14 (48.28) | 8 (36.36) |
| High | 15 (51.72) | 14 (63.64) |
| **PCNA** |  |  |
| Low | 12 (41.38) | 9 (40.91) |
| High | 17 (58.62) | 13 (50.09) |
| **CHEK1** |  |  |
| Low | 13 (44.83) | 10 (45.45) |
| High | 16 (55.17) | 12 (54.55) |
| **NEK2** |  |  |
| Low | 8 (27.59) | 11 (50.00) |
| High | 21 (72.41) | 11 (50.00) |
| **NCAPG2** |  |  |
| Low | 13 (44.83) | 8 (36.36) |
| High | 16 (55.17) | 14 (63.64) |
