# Supplementary material for: Transcriptomics-based liquid biopsy panel for early non-invasive identification of peritoneal recurrence and micrometastasis in locally advanced gastric cancer
Source: J Exp Clin Cancer Res. 2024 Jun 28;43:181. doi: 10.1186/s13046-024-03098-5 (PMC11212226; doi:10.1186/s13046-024-03098-5)
Supplement: Supplementary file 2 — Supplementary Material 2. [file 13046_2024_3098_MOESM2_ESM.docx]

**Supplementary Table 2 Clinical characteristics of training set and validation set of surgical resection specimen cohort[n(%)]**

| **Clinical characteristic** | **Training cohort**  **(N=196)** | **Validation cohort**  **(N=133)** | **P value** |
| --- | --- | --- | --- |
| **Gender** |  |  | 0.689 |
| Male | 121 (61.73) | 85 (63.91) |  |
| Female | 75 (38.24) | 48 (36.09) |  |
| **Age(years)** |  |  | 0.569 |
| ≤65 | 105 (53.57) | 67 (50.38) |  |
| ＞65 | 91 (46.43) | 66 (49.62) |  |
| **T stage** |  |  | 0.469 |
| T2/T3 | 9 ( 4.59) | 4 ( 3.01) |  |
| T4 | 187 (95.41) | 129 (96.99) |  |
| **N stage** |  |  | 0.711 |
| N0 | 38 (19.39) | 28 (21.05) |  |
| N+ | 158 (80.61) | 105 (78.95) |  |
| **Primary site** |  |  | 0.923 |
| Up 1/3 | 66 (33.67) | 47 (35.34) |  |
| Middle 1/3 | 46 (23.47) | 29 (21.80) |  |
| Lower 1/3 | 84 (42.86) | 57 (42.86) |  |
| **Tumor size(cm)** |  |  | 0.897 |
| ≤5 | 87 (44.39) | 60 (45.11) |  |
| ＞5 | 109 (55.61) | 73 (54.89) |  |
| **Histology** |  |  | 0.641 |
| None/Low | 146 (74.49) | 96 (72.18) |  |
| High/Median | 50 (25.51) | 37 (27.82) |  |
| **Lauren** |  |  | 0.850 |
| Diffuse/Mix type | 168 (85.71) | 113 (84.96) |  |
| Intestinal type | 28 (14.29) | 20 (15.04) |  |
| **Vascular invasion** |  |  | 0.319 |
| Yes | 59 (30.10) | 47 (35.34) |  |
| No | 137 (69.90) | 86 (64.66) |  |
| **Nerve invasion** |  |  | 0.840 |
| Yes | 123 (62.76) | 82 (61.65) |  |
| No | 73 (37.24) | 51 (38.35) |  |
| **BUB1** |  |  | 0.590 |
| Low | 75 (38.27) | 47 (35.34) |  |
| High | 121 (61.73) | 86 (64.66) |  |
| **CKS2** |  |  | 0.918 |
| Low | 77 (39.29) | 53 (39.85) |  |
| High | 119 (60.71) | 80 (60.15) |  |
| **PCNA** |  |  | 0.996 |
| Low | 81 (41.33) | 55 (41.35) |  |
| High | 115 (58.67) | 78 (58.65) |  |
| **CHEK1** |  |  | 0.791 |
| Low | 78 (39.80) | 51 (38.35) |  |
| High | 118 (60.20) | 82 (61.65) |  |
| **NEK2** |  |  | 0.409 |
| Low | 69 (35.20) | 41 (30.83) |  |
| High | 127 (64.80) | 92 (69.17) |  |
| **NCAPG2** |  |  | 0.935 |
| Low | 64 (32.65) | 44 (33.08) |  |
| High | 132 (67.35) | 89 (66.92) |  |
