# Supplementary material for: Transcriptomics-based liquid biopsy panel for early non-invasive identification of peritoneal recurrence and micrometastasis in locally advanced gastric cancer
Source: J Exp Clin Cancer Res. 2024 Jun 28;43:181. doi: 10.1186/s13046-024-03098-5 (PMC11212226; doi:10.1186/s13046-024-03098-5)
Supplement: Supplementary file 3 — Supplementary Material 3. [file 13046_2024_3098_MOESM3_ESM.docx]

**Supplementary Table 3 Clinical characteristics of the endoscopic biopsy specimen validation set cohort[n(%)]**

| **Clinical characteristic** | **Validation cohort**  **(N=103)** |
| --- | --- |
| **Gender** |  |
| Male | 66 (64.08) |
| Female | 37 (35.92) |
| **Age(years)** |  |
| ≤65 | 55 (53.40) |
| ＞65 | 48 (46.60) |
| **T stage** |  |
| T2/T3 | 4 ( 3.88) |
| T4 | 99 (96.12) |
| **N stage** |  |
| N0 | 23 (22.33) |
| N+ | 80 (77.67) |
| **Primary site** |  |
| Up 1/3 | 37 (35.92) |
| Middle 1/3 | 20 (19.42) |
| Lower 1/3 | 46 (44.66) |
| **Tumor size(cm)** |  |
| ≤5 | 45 (43.69) |
| ＞5 | 58 (56.31) |
| **Histology** |  |
| None/Low | 79 (76.70) |
| High/Median | 24 (23.30) |
| **Lauren** |  |
| Diffuse/Mix type | 88 (85.44) |
| Intestinal type | 15 (14.56) |
| **Vascular invasion** |  |
| Yes | 34 (33.01) |
| No | 69 (66.99) |
| **Nerve invasion** |  |
| Yes | 62 (60.19) |
| No | 41 (39.81) |
| **BUB1** |  |
| Low | 35 (33.98) |
| High | 68 (66.02) |
| **CKS2** |  |
| Low | 41 (39.81) |
| High | 62 (60.19) |
| **PCNA** |  |
| Low | 43 (41.75) |
| High | 60 (58.25) |
| **CHEK1** |  |
| Low | 40 (38.83) |
| High | 63 (61.17) |
| **NEK2** |  |
| Low | 31 (30.10) |
| High | 72 (69.90) |
| **NCAPG2** |  |
| Low | 36 (34.95) |
| High | 67 (65.05) |
