# Supplementary material for: Transcriptomics-based liquid biopsy panel for early non-invasive identification of peritoneal recurrence and micrometastasis in locally advanced gastric cancer
Source: J Exp Clin Cancer Res. 2024 Jun 28;43:181. doi: 10.1186/s13046-024-03098-5 (PMC11212226; doi:10.1186/s13046-024-03098-5)
Supplement: Supplementary file 4 — Supplementary Material 4. [file 13046_2024_3098_MOESM4_ESM.docx]

**Supplementary Table 4 Clinical characteristics of peripheral blood specimen cohorts in training set and validation set[n(%)]**

| **Clinical characteristic** | **Training cohort**  **(N=120)** | **Validation cohort**  **(N=123)** | **P value** |
| --- | --- | --- | --- |
| **Gender** |  |  | 0.800 |
| Male | 77 (64.17) | 77 (62.60) |  |
| Female | 43 (35.83) | 46 (37.40) |  |
| **Age(years)** |  |  | 0.952 |
| ≤65 | 61 (50.83) | 63 (51.22) |  |
| ＞65 | 59 (49.17) | 60 (48.78) |  |
| **T stage** |  |  | 0.972 |
| T2/T3 | 4 ( 3.33) | 4 ( 3.25) |  |
| T4 | 116 (96.67) | 119 (96.75) |  |
| **N stage** |  |  | 0.920 |
| N0 | 26 (21.67) | 26 (21.14) |  |
| N+ | 94 (78.33) | 97 (78.86) |  |
| **Primary site** |  |  | 0.861 |
| Up 1/3 | 42 (35.00) | 39 (31.71) |  |
| Middle 1/3 | 31 (25.83) | 33 (26.83) |  |
| Lower 1/3 | 47 (39.17) | 51 (41.46) |  |
| **Tumor size(cm)** |  |  | 0.637 |
| ≤5 | 52 (43.33) | 57 (46.34) |  |
| ＞5 | 68 (56.67) | 66 (53.66) |  |
| **Histology** |  |  | 0.864 |
| None/Low | 88 (73.33) | 89 (72.36) |  |
| High/Median | 32 (26.67) | 34 (27.64) |  |
| **Lauren** |  |  | 0.917 |
| Diffuse/Mix type | 103 (85.83) | 105 (85.37) |  |
| Intestinal type | 17 (14.17) | 18 (14.63) |  |
| **Vascular invasion** |  |  | 0.693 |
| Yes | 41 (34.17) | 45 (36.59) |  |
| No | 79 (65.83) | 78 (63.41) |  |
| **Nerve invasion** |  |  | 0.777 |
| Yes | 73 (60.83) | 77 (62.60) |  |
| No | 47 (39.17) | 46 (37.40) |  |
| **BUB1** |  |  | 0.889 |
| Low | 42 (35.00) | 42 (34.15) |  |
| High | 78 (65.00) | 81 (65.85) |  |
| **CKS2** |  |  | 0.773 |
| Low | 49 (40.83) | 48 (39.02) |  |
| High | 71 (59.17) | 75 (60.98) |  |
| **PCNA** |  |  | 0.921 |
| Low | 49 (40.83) | 51 (41.46) |  |
| High | 71 (59.17) | 72 (58.54) |  |
| **CHEK1** |  |  | 0.984 |
| Low | 46 (38.33) | 47 (38.21) |  |
| High | 74 (61.67) | 76 (61.79) |  |
| **NEK2** |  |  | 0.899 |
| Low | 37 (30.83) | 37 (30.08) |  |
| High | 83 (69.17) | 86 (69.92) |  |
| **NCAPG2** |  |  | 1.000 |
| Low | 40 (33.33) | 41 (33.33) |  |
| High | 80 (66.67) | 82 (66.67) |  |
