# Supplementary material for: Transcriptomics-based liquid biopsy panel for early non-invasive identification of peritoneal recurrence and micrometastasis in locally advanced gastric cancer
Source: J Exp Clin Cancer Res. 2024 Jun 28;43:181. doi: 10.1186/s13046-024-03098-5 (PMC11212226; doi:10.1186/s13046-024-03098-5)
Supplement: Supplementary file 5 — Supplementary Material 5. [file 13046_2024_3098_MOESM5_ESM.docx]

**Supplementary Table 5 Clinical characteristics of peripheral blood specimen cohorts in training set and validation set[n(%)]**

| **Clinical characteristic** | **P0CY1 cohort**  **(N=66)** | **P1CY1 cohort**  **(N=95)** |
| --- | --- | --- |
| **Gender** |  |  |
| Male | 40 (60.61) | 57 (60.00) |
| Female | 26 (39.39) | 38 (40.00) |
| **Age(years)** |  |  |
| ≤65 | 35 (53.03) | 50 (52.63) |
| ＞65 | 31 (46.97) | 45 (47.37) |
| **T stage** |  |  |
| T2/T3 | 1 ( 1.52) | 1 ( 1.05) |
| T4 | 65 (98.48) | 94 (98.95) |
| **N stage** |  |  |
| N0 | 13 (19.70) | 19 (20.00) |
| N+ | 53 (80.30) | 76 (80.00) |
| **Primary site** |  |  |
| Up 1/3 | 20 (30.30) | 37 (38.95) |
| Middle 1/3 | 13 (19.70) | 17 (17.89) |
| Lower 1/3 | 33 (50.00) | 41 (43.16) |
| **Tumor size(cm)** |  |  |
| ≤5 | 34 (51.52) | 45 (47.37) |
| ＞5 | 32 (48.48) | 50 (52.63) |
| **Histology** |  |  |
| None/Low | 50 (75.76) | 73 (76.84) |
| High/Median | 16 (24.24) | 22 (23.16) |
| **Lauren** |  |  |
| Diffuse/Mix type | 61 (92.42) | 82 (86.32) |
| Intestinal type | 5 ( 7.58) | 13 (13.68) |
| **Vascular invasion** |  |  |
| Yes | 22 (33.33) | 28 (29.47) |
| No | 44 (66.67) | 67 (70.53) |
| **Nerve invasion** |  |  |
| Yes | 40 (60.61) | 58 (61.05) |
| No | 26 (39.39) | 37 (38.95) |
| **BUB1** |  |  |
| Low | 22 (33.33) | 35 (36.84) |
| High | 44 (66.67) | 60 (63.16) |
| **CKS2** |  |  |
| Low | 23 (34.85) | 31 (32.63) |
| High | 43 (65.15) | 64 (67.37) |
| **PCNA** |  |  |
| Low | 29 (43.94) | 41 (43.16) |
| High | 37 (56.06) | 54 (56.84) |
| **CHEK1** |  |  |
| Low | 26 (39.39) | 39 (41.05) |
| High | 40 (60.61) | 56 (58.95) |
| **NEK2** |  |  |
| Low | 18 (27.27) | 29 (30.53) |
| High | 48 (72.73) | 66 (69.47) |
| **NCAPG2** |  |  |
| Low | 23 (34.85) | 31 (32.63) |
| High | 43 (65.15) | 64 (67.37) |
