# Supplementary material for: Transcriptomics-based liquid biopsy panel for early non-invasive identification of peritoneal recurrence and micrometastasis in locally advanced gastric cancer
Source: J Exp Clin Cancer Res. 2024 Jun 28;43:181. doi: 10.1186/s13046-024-03098-5 (PMC11212226; doi:10.1186/s13046-024-03098-5)
Supplement: Supplementary file 7 — Supplementary Material 7. [file 13046_2024_3098_MOESM7_ESM.docx]

**Supplementary Table 7 Multifactorial logistic regression analysis of 6 candidate mRNAs influencing the occurrence of PM in GC patients**

| **Surgical resection specimens--training cohort** | | | |
| --- | --- | --- | --- |
| **mRNA** | **OR** | **95%CI** | **P value** |
| BUB1 (High vs. Low) | 9.534 | 2.384-38.121 | 0.001 |
| CKS2 (High vs. Low) | 5.647 | 1.616-19.741 | 0.007 |
| PCNA (High vs. Low) | 7.968 | 2.279-27.860 | 0.001 |
| CHEK1 (High vs. Low) | 5.567 | 1.672-18.538 | 0.005 |
| NEK2 (High vs. Low) | 5.464 | 1.440-20.738 | 0.013 |
| NCAPG2 (High vs. Low) | 3.894 | 1.171-12.956 | 0.027 |
| **Surgical resection specimens--validation cohort** | | | |
| **mRNA** | **OR** | **95%CI** | **P value** |
| BUB1 (High vs. Low) | 4.882 | 1.099-21.691 | 0.037 |
| CKS2 (High vs. Low) | 5.744 | 1.329-24.829 | 0.019 |
| PCNA (High vs. Low) | 10.411 | 1.966-55.118 | 0.006 |
| CHEK1 (High vs. Low) | 5.316 | 1.161-24.329 | 0.031 |
| NEK2 (High vs. Low) | 9.052 | 1.091-75.122 | 0.041 |
| NCAPG2 (High vs. Low) | 3.643 | 1.080-16.595 | 0.045 |
| **Gastroscopy biopsy specimens--validation cohort** | | | |
| **mRNA** | **OR** | **95%CI** | **P value** |
| BUB1 (High vs. Low) | 6.764 | 1.092-41.899 | 0.040 |
| CKS2 (High vs. Low) | 3.353 | 1.081-16.504 | 0.037 |
| PCNA (High vs. Low) | 20.380 | 2.123-195.627 | 0.009 |
| CHEK1 (High vs. Low) | 8.619 | 1.333-55.738 | 0.024 |
| NEK2 (High vs. Low) | 7.417 | 1.134-65.919 | 0.032 |
| NCAPG2 (High vs. Low) | 3.830 | 1.073-20.053 | 0.012 |
| **Peripheral blood specimens--training cohort** | | | |
| **mRNA** | **OR** | **95%CI** | **P value** |
| BUB1 (High vs. Low) | 3.636 | 1.803-16.471 | 0.044 |
| CKS2 (High vs. Low) | 4.424 | 1.081-19.553 | 0.039 |
| PCNA (High vs. Low) | 18.860 | 2.163-164.468 | 0.008 |
| CHEK1 (High vs. Low) | 4.556 | 1.165-21.507 | 0.035 |
| NEK2 (High vs. Low) | 9.152 | 1.088-77.009 | 0.042 |
| NCAPG2 (High vs. Low) | 3.326 | 1.017-15.426 | 0.025 |
| **Peripheral blood specimens--validation cohort** | | | |
| **mRNA** | **OR** | **95%CI** | **P value** |
| BUB1 (High vs. Low) | 3.767 | 1.044-16.814 | 0.042 |
| CKS2 (High vs. Low) | 5.329 | 1.218-23.325 | 0.026 |
| PCNA (High vs. Low) | 9.959 | 1.877-52.852 | 0.007 |
| CHEK1 (High vs. Low) | 5.146 | 1.129-23.460 | 0.034 |
| NEK2 (High vs. Low) | 9.165 | 1.102-76.182 | 0.040 |
| NCAPG2 (High vs. Low) | 3.539 | 1.078-16.090 | 0.030 |
