# Supplementary material for: Transcriptomics-based liquid biopsy panel for early non-invasive identification of peritoneal recurrence and micrometastasis in locally advanced gastric cancer
Source: J Exp Clin Cancer Res. 2024 Jun 28;43:181. doi: 10.1186/s13046-024-03098-5 (PMC11212226; doi:10.1186/s13046-024-03098-5)
Supplement: Supplementary file 8 — Supplementary Material 8. [file 13046_2024_3098_MOESM8_ESM.docx]

**Supplementary Table 8 Multifactorial logistic regression analysis influencing the occurrence of PM in patients with GC**

| **Surgical resection specimens--training cohort** | | | |
| --- | --- | --- | --- |
| **Variables** | **OR** | **95%CI** | **P value** |
| T stage (T4 vs. T2/T3) | 7.453 | 1.569-35.401 | 0.012 |
| Tumour size (≥5cm vs. ＜5cm) | 5.780 | 1.183-30.873 | 0.021 |
| Vascular invasion (Yes vs. No) | 4.124 | 1.078-15.773 | 0.038 |
| 6-mRNA panel (High vs. Low) | 6.634 | 1.325-28.943 | 0.015 |
| **Surgical resection specimens--validation cohort** | | | |
| **Variables** | **OR** | **95%CI** | **P value** |
| T stage (T4 vs. T2/T3) | 8.706 | 1.438-42.443 | 0.001 |
| Tumour size (≥5cm vs. ＜5cm) | 4.824 | 1.147-13.543 | 0.012 |
| Vascular invasion (Yes vs. No) | 3.720 | 1.134-10.617 | 0.011 |
| 6-mRNA panel (High vs. Low) | 6.555 | 1.250-32.546 | 0.009 |
| **Gastroscopy biopsy specimens--validation cohort** | | | |
| **Variables** | **OR** | **95%CI** | **P value** |
| T stage (T4 vs. T2/T3) | 10.348 | 1.721-47.647 | 0.005 |
| Tumour size (≥5cm vs. ＜5cm) | 5.098 | 1.332-19.651 | 0.011 |
| Vascular invasion (Yes vs. No) | 3.856 | 1.173-10.432 | 0.035 |
| 6-mRNA panel (High vs. Low) | 7.895 | 1.567-39.663 | 0.010 |
| **Peripheral blood specimens--training cohort** | | | |
| **Variables** | **OR** | **95%CI** | **P value** |
| T stage (T4 vs. T2/T3) | 8.789 | 1.564-35.787 | 0.006 |
| Tumour size (≥5cm vs. ＜5cm) | 4.658 | 1.134-13.368 | 0.023 |
| Vascular invasion (Yes vs. No) | 2.643 | 1.021-10.527 | 0.046 |
| 6-mRNA panel (High vs. Low) | 6.955 | 1.431-28.530 | 0.010 |
| **Peripheral blood specimens--validation cohort** | | | |
| **Variables** | **OR** | **95%CI** | **P value** |
| T stage (T4 vs. T2/T3) | 11.542 | 2.158-49.657 | 0.001 |
| Tumour size (≥5cm vs. ＜5cm) | 5.643 | 1.457-21.552 | 0.021 |
| Vascular invasion (Yes vs. No) | 3.066 | 1.174-13.570 | 0.032 |
| 6-mRNA panel (High vs. Low) | 8.644 | 1.873-37.404 | 0.009 |
