# Supplementary material for: Transcriptomics-based liquid biopsy panel for early non-invasive identification of peritoneal recurrence and micrometastasis in locally advanced gastric cancer
Source: J Exp Clin Cancer Res. 2024 Jun 28;43:181. doi: 10.1186/s13046-024-03098-5 (PMC11212226; doi:10.1186/s13046-024-03098-5)
Supplement: Supplementary file 9 — Supplementary Material 9. [file 13046_2024_3098_MOESM9_ESM.docx]

**Supplementary Figure legends**


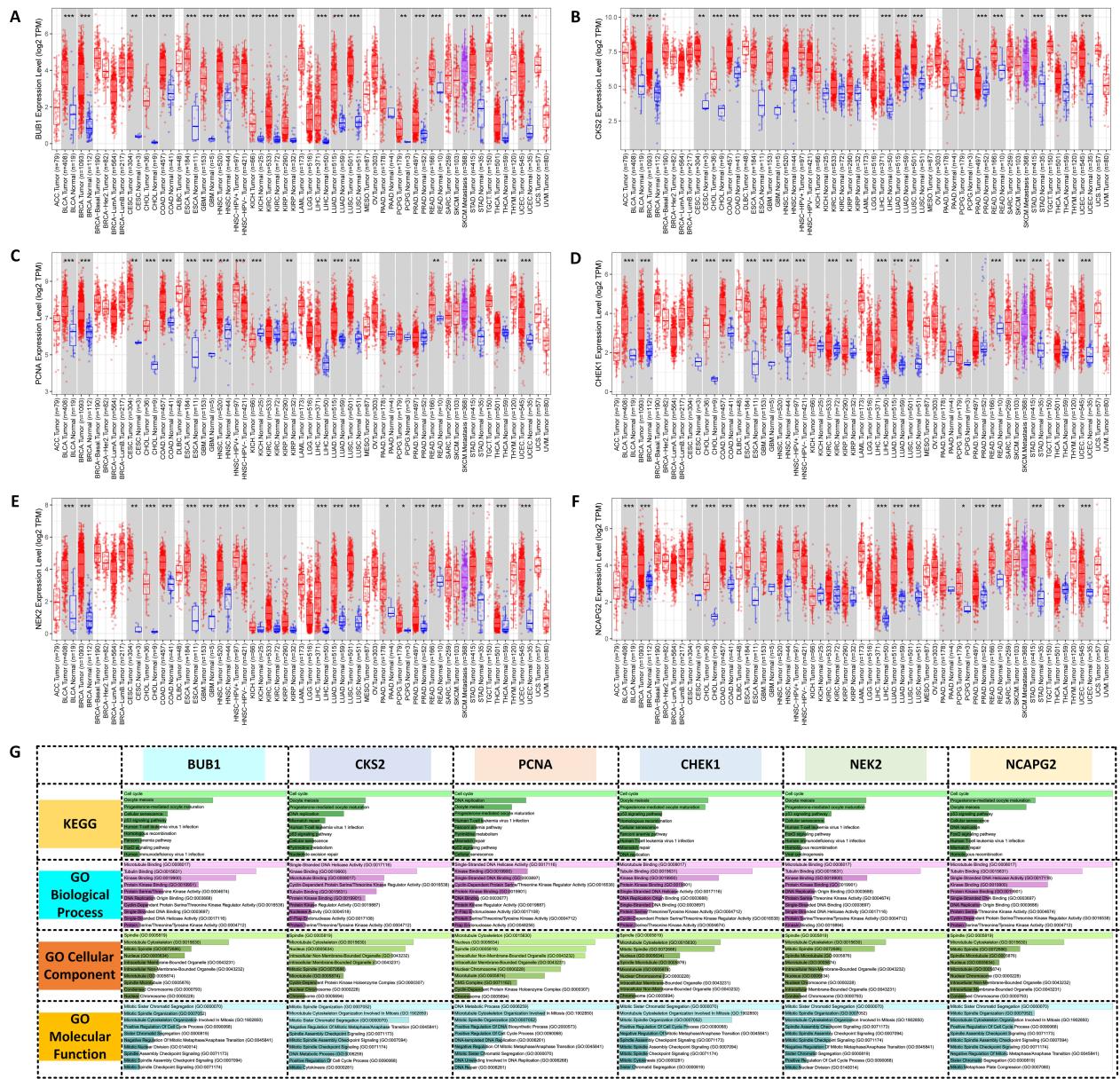


**Supplementary Figure 1 Expression of 6 genes in pan-cancer and pathway enrichment.** A, Expression of BUB1 in pan-cancer; B, Expression of CKS2 in pan-cancer; C, Expression of PCNA in pan-cancer; D, Expression of CHEK1 in pan-cancer; E, Expression of NEK2 in pan-cancer; F, Expression of NCAPG2 in pan-cancer; G, Expression of 6 genes in KEGG and GO pathways. enrichment analysis.


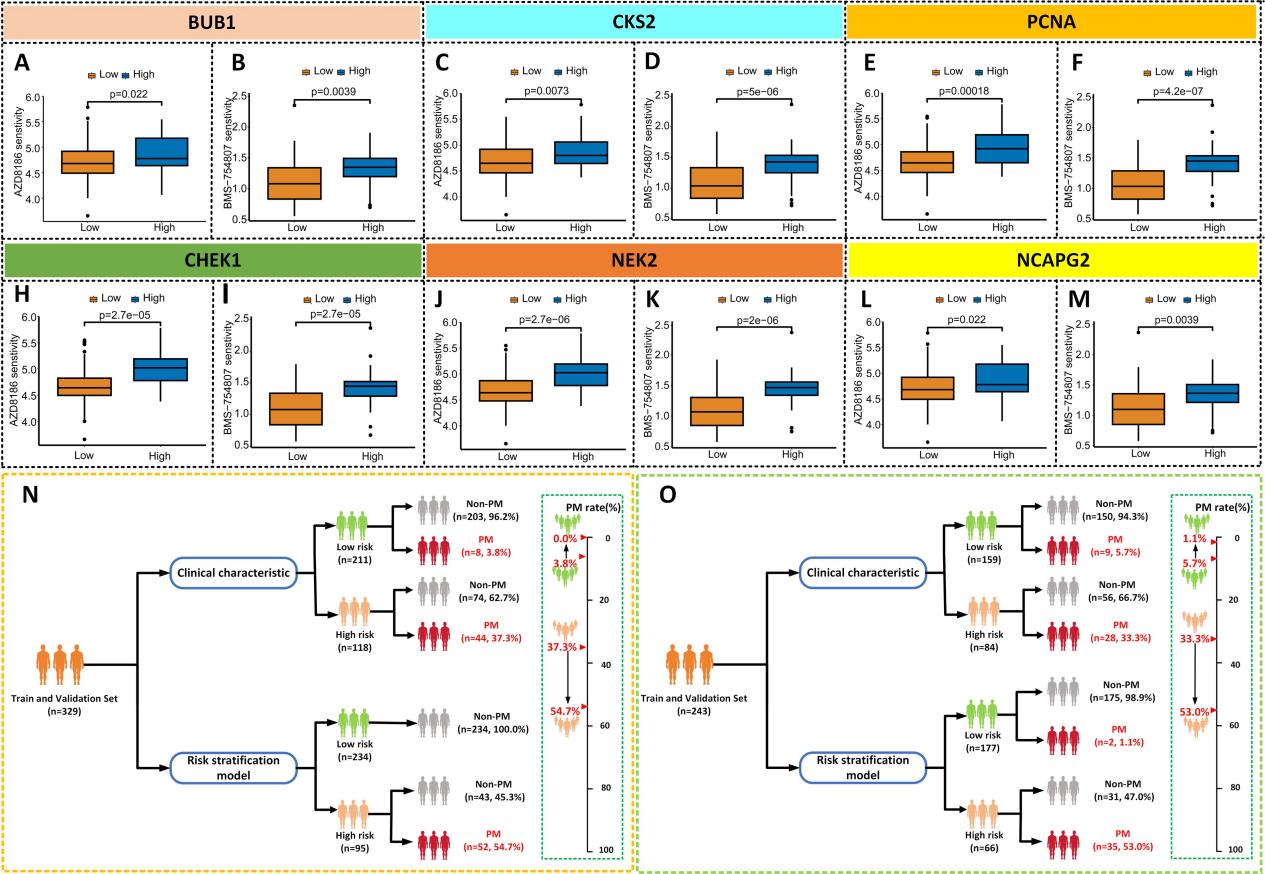


**Supplementary Figure 2 6-gene drug sensitivity analyses and the clinical benefit of the prediction model.** A, BUB1 expression versus AZD8186 drug sensitivity analysis; B, BUB1 expression versus BMS-754807 drug sensitivity analysis; C, CKS2 expression versus AZD8186 drug sensitivity analysis; D, CKS2 expression versus BMS-754807 drug sensitivity analysis; E, PCNA expression versus AZD8186 drug sensitivity analysis; F, PCNA expression versus BMS-754807 drug sensitivity analysis; G, CHEK1 expression versus AZD8186 drug sensitivity analysis; H, CHEK1 expression versus BMS-754807 drug sensitivity analysis; I, NEK2 expression versus AZD8186 drug sensitivity analysis; J, NEK2 expression versus BMS-754807 drug sensitivity analysis; K, BUB1 expression versus AZD8186 drug sensitivity analysis; L, BUB1 expression versus BMS-754807 drug sensitivity analysis; M, Clinical benefit of surgical ex vivo fresh-frozen specimen cohort; N, Peripheral blood specimen Clinical benefit profile of the cohort.
